# Investigation of Propolis’ Effect on Thiobarbituric Acid Reactive Substances and Anti-Oxidant Enzyme Levels of Hippocampus in Diabetic Rats Induced by Streptozotocin

**DOI:** 10.3889/oamjms.2015.031

**Published:** 2015-03-08

**Authors:** Burcu Köksal, Memet Hanifi Emre, Alaadin Polat

**Affiliations:** *Inonu University, Faculty of Medicine, Department of Physiology, 44280 Malatya, Turkey*

**Keywords:** Diabetes, propolis, anti-oxidative enzymes, streptozotocine, rats

## Abstract

**BACKGROUND:**

Propolis is an organic resinous viscous substance collected from flower bud and plant sprig by bees. Propolis has a potential treatment agent for oxidative damage caused by diabetes in hippocampus due to its flavonoid and phenolic content.

**AIM:**

In this study effect of propolis on thiobarbituric acid reactive substances and anti-oxidative enzyme levels of hippocampus in diabetic rats induced by streptozotocin was investigated.

**MATERIALS AND METHODS:**

The study involved measuring levels of SOD, CAT, GSH-Px and TBARs in hippocampus tissue of STZ-induced diabetic rats (Adult Male Sprague Dawley rats) after applying propolis for one month. The subjects of the study were composed of 51 rats randomly assigned to four groups (Control, STZ, P+STZ and STZ+P). For analysis of data, Kruskal Wallis Test was utilized.

**RESULTS:**

The findings of the study showed that there were no significant difference in the levels of TBARS, SOD, CAT and GSH-Px of hippocampus across the groups.

**CONCLUSION:**

Propolis application in four-week duration does not have effect on TBARS, SOD, CAT and GSH-Px levels of hippocampus of diabetic rats. These findings mean that more time for observing oxidative harms on hippocampus is needed.

## Introduction

Diabetes is a serious disease characterized by insufficient insulin secretion, insulin activity deficiency and hyperglycemia, and its estimated prevalence is pretty high (5.4% in 2025) in society [1, 2, 3]. At the same time it causes to serious problems involving blindness, pancreatic cancer and cognitive impairment [4-6]. Moreover the molecular level effects of diabetes also exist and they involve increase in oxidative agent levels and deficit in anti-oxidative systems [7, 8]. In particular hyperglycemia in diabetes causes increase of oxidative radicals and then the radicals lead to diabetic complications [7]. Especially these oxidative radicals lead to structural defects of membranes and proteins [9]. When N-nitroso derivate to make diabetes model was applied and then products of the application was observed, it was seen that application of streptozotocin (N-nitroso derivate) produced oxidative radicals and demolished Langerhans islets in pancreas [10]. Also it was shown that increase in glucose levels was associated with increase in levels of oxidative radicals [8]. Increased oxidative radicals stimulate damage in diabetic individuals and the damages results in tissue injury and membrane damage [11, 12].

In the researches thiobarbituric acid reactive substances (TBARs), superoxide dismutase (SOD), catalase (CAT) and glutathione peroxidase (GSH-px) are frequently studied enzymes and molecules in anti-oxidative studies of tissues [13, 14]. Hippocampus tissue which is important in learning and memory functions of diabetic individuals is also affected by oxidative stress caused by diabetes, certain neuronal death in CA1, CA3 and DG regions of hippocampus and oxidative stress in hippocampus are reported [15, 16]. Measuring acid reactive substances (TBARs), superoxide dismutase (SOD), catalase (CAT) and glutathione peroxidase (GSH-px) level in hippocampus after using a potential treatment agent might provide molecular evidence for effect of the agent. Propolis has a potential treatment agent for oxidative damage due to its flavonoid and phenolic content [17, 18]. Since flavonoids and phenols have high anti-oxidative effects [19]. Propolis is an organic resinous viscous substance collected from flower bud and plant sprig by bees and its content involves phenolic acids, and their esters, terpenoids, steroids, caffeic acid phenethyl ester (CAPE) and various amino-acids [20-22]. Propolis represents anti-oxidative activity toward superoxide anion and also it decreases lipid peroxidation by increasing SOD activity [23]. In addition propolis involves zinc (Zn) and hence it contributes effect of anti-oxidative enzyme activity due to involvement of Zn in anti-oxidative enzymes as a co-factor [24]. Especially quercetin content of propolis was found to be an effective agent in inhibiting oxidative damage in hippocampus [25].

Based on the potential of propolis for changing levels of thiobarbituric acid reactive substances and anti-oxidative enzyme levels of hippocampus when diabetes is in case, investigation of potential effects of propolis on the levels of thiobarbituric acid reactive substances and anti-oxidative enzyme levels of hippocampus of diabetic rats was purposed.

## Materials and Method

### Animals

In this study, 51 Adult Male Spraque Dawley rats (2.5 months) were involved (Mean Weight = 266 ± 30g). They were taken from The Research Center of Experimental Animals in Inonu University. The rats were hold in a ventilated room during all days of the study. The room temperature was constant at 20ºC ± 2 and lightening was adjusted as 12-hour light and 12-hour dark periods. For ethical approval, the study was evaluated by Ethic Committee of Inonu University for Medical Research and the committee approved the study with record number; 2010/36.

### Experimental Groups and Processes

The rats were randomly assigned into four groups of the study and they were put into cages for five rats. After the assignment, non-diabetic rats (n = 9) were excluded from the study. The final groups and number of the rats for each group are represented below:

1. Group: Control (n=14);
2. Group: STZ (n=12);
3. Group: Propolis before STZ application (P+STZ) (n=12);
4. Group: STZ application before Propolis (STZ+P) (n=13).

In the control group, ordinary pellet feed was provided to the rats and they drank ordinary tap water when they needed. In the STZ group, dissolved STZ in distillated water was intraperitonally injected into the rats as just one-dose after one-day fasting (45 mg/kg) [26-28]. In another group (P+STZ), propolis (0.012 mg/ml) was applied to the rats by adding its solution to drinking water two days before STZ injection and then STZ was intraperitonally injected as in STZ group. In following days propolis was applied for 30 days in drinking water [29-31]. Reason of applying propolis two days before STZ application is to check its protective effect. In the STZ+P group, STZ was intraperitonally injected into the rats and three days after STZ application, freshly prepared propolis (0.012 mg/ml) was applied as a solution in drinking water for 30 days [29-31]. Four weeks for the study was found enough to study brain changes since Huang, Gao, Yang, Lin and Lei saw brain abnormalities four weeks after STZ application [32].

For detecting diabetic situation, one drop of blood was taken from the tails of the rats. For taking blood apex of the tails was cut and blood glucose levels were measured at the time of 72 hours after STZ application. For measurement, On Call Plus blood glucose meter (*ACON international*) was utilized [33]. 72-hour period was enough to see diabetic conditions in rats after STZ application.

### Preparation of Propolis

In this study propolis of chestnut honey was obtained from Zonguldak Honey Association in Northwest Region of Turkey. Propolis was held in ordinary room conditions (dark and humid-free). Propolis was prepared and applied as described in the literature [29, 30, 34]. Amount of the propolis taken by the rats in the groups was accepted similar in terms of group average since our unit of analysis was not individual rats. We compared the groups by using statistics.

### Preparation of Hippocampus Tissue for Oxidative Stress Studies

After taking the hippocampus tissues, they washed in ice-cold phosphate buffered saline (PBS) (50 mM), and then they were put into deepfreeze (under -80°C) for holding them safe up to the study time in lab [35]. At the time of study, they were taken out from deepfreeze and weighed. For preparing the tissues in studying TBARs, SOD, CAT and GSH-Px, Tris–HCl buffer (2 ml, pH = 7.4) was used, Tris-HCl buffer was added onto the tissues in glass tubes. Then the tubes were placed into plastic cover filled with ice and homogenization of the tissues was done for 2 min in 16000 rpm. The final volume of the homogenate was completed to 3 ml by adding Tris-HCl buffer. Homogenate was stirred with vortex for 1-2 min and 1 ml of homogenate was taken for extra needs (such as failure in process or technical obstacles) in the study, and 0.5 ml homogenate was put into Ependorf tubes for TBARs measurements. The remaining homogenate (1.5 ml) was centrifuged in 4000 rpm for 15 min. (+4°C) for SOD, GSH-Px and CAT studies and then supernatant was taken from centrifuged homogenate and reserved in deep freeze [36, 37].

### SOD Activity Level Analysis

SOD enzyme activity was measured by the method suggested by Sun et al. [38]. In the method, reduction reaction of superoxide radicals by nitrobluetetrazolium is utilized. Xanthine/Xanthine oxidase is used as producer of superoxide radicals and then reaction between superoxide radicals and tetrazolium salts produces chromogenic formazone dye. The dye gives maximum absorbance at 560 nm. If SOD is existent in reaction, formation of formazone is prevented by SOD and blue-purple color is observed. The findings of the spectrophotometer analysis are reported as U/mg protein.

### CAT Activity Level Analysis

CAT activity was measured by considering Aebi’s method [39]. If H_2_O_2_ is added to the solution of CAT enzyme, the CAT divides it into H_2_O and O_2_. This reaction gives maximum absorbance in 240nm and then decreasing absorbance is observed in UV spectrophotometer. This decrease is a result of CAT activity. For measuring CAT activity, decrease in absorbance was recorded in 240 nm for 5 min after adding sample to phosphate buffer adjusted to 0.500 optic density values by H_2_O_2_. The findings of the spectrophotometer analysis are reported as K/g protein.

### GSH-Px Activity Level Analysis

GSH-Px activity was studied by Paglia and Valentine’s method [40]. The method is based on absorbance decrease (340 nm) caused by removal of NADPH molecules by glutathione reductase. After the reaction glutathione and NADP^+^ remain in the reaction site. The findings of the spectrophotometer analysis are reported as U/mg protein.

### TBARs Level Analysis

TBARs analysis was done by Uchiyama and Mihara’s method [41]. In the method N-butanole is added to pink homogenate formed by reaction of malonedialdehide (MDA) and tiobartbutiric acid at 95°C. The reaction products are observed in 535 and 520 nm by spectrophotometer. The results are reported as nmol/g tissue.

### Lowry Protein Method

The enzyme activity regarding GSH-Px and SOD represented above should be investigated by using rate of enzymes in total protein. So there was a need to determine total protein level of tissues. Total protein measurements were conducted by modified Lowry method [42]. The method is based on formation of Cu-protein complex under alkaline conditions and formation of blue color by the reaction between Folin-Ciocalteu and acid in indicator. In the method formation of dark blue is seen and maximum absorbance is seen at 750 nm. Total protein amount in the tissues is reported as µg/ml.

### Statistical Analysis

For the purpose of the study, Kruskal-Wallis technique (by SPSS Program) was utilized to compare data across the groups in terms of the variables. Main reason for selecting the technique was that normality assumption and sample size for parametric statistics were not provided enough. For the comparisons, .05 was set for making type I error [43].

## Results

The results of the analysis showed that STZ application increased blood glucose levels of rats over 200 mg/dl (Control = 88 ± 11, STZ = 313 ± 83, P+STZ = 320 ± 59, STZ+P = 301 ± 56) and weights of the rats in STZ applied groups were lower than control group. These are accepted as criteria for being diabetic. Then TBARs, GSH-Px, SOD and CAT levels were measured after one-month treatment. The descriptive findings about TBARs, GSH-Px, SOD and CAT are represented in Table 1.

**Table 1:** TBARs, GSH-Px, SOD and CAT levels of rats’ hippocampus after the propolis treatment.

Group	TBARs (nmol/g)	GSH-Px (U/mg)	SOD (U/mg)	CAT (K/g)

	Mean/SD	Mean/SD	Mean/SD	Mean/SD
Control (n=14)	0.30 ± 0.09	24.50 ± 4.79	15.51 ± 3.19	1.82 ± 0.48

STZ (n=12)	0.31 ± 0.09	28.75 ± 7.92	18.65 ± 7.30	1.97 ± 0.48

P+STZ (n=12)	0.39 ± 0.11	31.23 ± 7.25	23.12 ± 6.20	1.29 ± 0.66

STZ+P (n=13)	0.33 ± 0.04	27.60 ± 6.91	21.22 ± 5.25	2.31 ± 0.82

As seen in Table 1, highest level of TBARs is seen in P+STZ while the lowest level is seen in control group. For GSH-Px activity, highest level is recoded for P+STZ group while the lowest level is for control group. In SOD activity, control group represent lowest enzyme activity while P+STZ group represents highest level of activity. But CAT activity represents different result that highest activity is seen in STZ+P while the lowest activity level is seen in P+STZ group. When looked at the comparisons across the groups, it is seen that there are no significant differences between the groups in terms of TBARs, GSH-Px, SOD and CAT activities (see Table 2).

**Table 2:** Inferential statistical analysis results for comparing TBARs, GSH-Px, SOD and CAT levels of rats’ hippocampus after the propolis treatment across the groups.

Groups for Comparions	Dependent Variables	Degrees of freedom	Chi-square	*p*
Control (n=14)	TBARs (nmol/g)	3	3.87	0.28
	
STZ (n=12)	SOD (U/mg)	3	6.40	0.09
	
P+STZ (n=12)	CAT (K/g)	3	5.98	0.11
	
STZ+P (n=13)	GSH-Px (U/mg)	3	3.46	0.33

## Discussion

The results of the study supported the conclusion that STZ-induced diabetes increases blood glucose level of the rats. The reason of the increase in blood glucose levels is that STZ enter into β cells of pancreas islets and then it causes toxicity and cell destroys. Toxicity and cell destroys block insulin production and secretion, and finally permanent diabetes occurs [44]. Insulin dependent glucose transporters in diabetes are not able to carry required amount of glucose into the cells due to lack of insulin. Hence, blood glucose levels of rats increase [44]. Increased blood glucose levels lead to oxidative stress [7, 8]. Based on the diabetic situation and related oxidative stress, it is expected in this study that hippocampus of the rats is affected by oxidative stress caused by diabetes and we should see changes in oxidative and anti-oxidative levels. For testing this expectation, TBARs, SOD, CAT and GSH-Px levels were compared across the groups, but the findings were not in line with the expectation since there were no significant differences between the groups in terms of TBARs, SOD, CAT and GSH-Px. These findings should be explained with care because there is a difference between control group and STZ-applied groups in terms of TBARs in spite of non-significant difference. The similar situation is valid for GSH-Px and SOD. However non-significance of the difference brings time issue into the center of discussion. In the literature, it is shown that lipid peroxidation in rat brain needs time [45]. The findings of the previous studies explained that deficits in brain by chronic diabetes can be seen after 12 week-period [46-50].

Some of the studies support the conclusion that GSH-Px, CAT and SOD levels are not different from control group in diabetes studies while the others advocate that GSH-Px, CAT and SOD levels are different from control group in diabetes studies [45, 51-53]. We advocate second conclusion but there is a need to make the applications for more time. In the following studies there is a need to compare the groups after making the applications for longer time interval. As another suggestion application of propolis should be done by gavage into the stomach.
